# Acute hemolytic transfusion reaction induced prolonged renal injury in an obstetric patient: A case report

**DOI:** 10.1016/j.amsu.2022.103383

**Published:** 2022-02-12

**Authors:** Mohammad Alsultan

**Affiliations:** Department of Nephrology, Al Assad and Al Mouwasat University Hospitals, Damascus University- Faculty of Medicine, Damascus, Syria

**Keywords:** Red blood cell (RBC) transfusion, Acute hemolytic transfusion reaction (AHTR), Acute kidney injury (AKI), Case report

## Abstract

A red blood cell (RBC) transfusion can be fatal if an acute hemolytic transfusion reaction (AHTR) occurs. In the past, ABO-incompatible blood transfusions were the most common cause of hemolysis-associated acute kidney injury (AKI); however, these are now rare due to improving blood banking practices. A 29-year- old obstetric female of blood group A positive was admitted due to anuric AKI and intravascular hemolysis after receiving an incompatible transfusion of blood group AB positive. The patient displayed a classic triad of symptoms a few minutes after the transfusion and fortunately, the infant was saved by the performance of an immediate cesarean section. The patient required four sessions of hemodialysis during their hospital stay due to severe uremia and acute pulmonary edema. Kidney function improved very slowly and returned to near normal after six weeks.

This case was the second obstetric patient; admitted to our hospital; in the past few months with prolonged AKI induced by an ABO-incompatible blood transfusion. Complications arising from a RBC transfusion can be exhausting for the patient and medical staff and require a long hospital stay and high costs. This demonstrates the need for medical staff to reserve blood transfusions for obvious indications, to repeat the blood type, and to confirm the recorded compatibility of the patient and the blood unit before transfusion. Also, medical staff should always monitor the patient's symptoms during the transfusion process, to recognize these severe conditions and to administer effective treatments as soon as possible.

## Highlights

• Acute hemolytic transfusion reaction (AHTR) can induce a prolonged and severe acute kidney injury (AKI) that needs a long hospital stay and high costs.
• Medical staff should reserve blood transfusions for obvious indications and should confirm the recorded compatibility of the patient and the blood unit before transfusion.
• Clinicians should monitor symptoms during the transfusion, to early recognize these conditions and to administer effective treatments.

## 1 Introduction

Although a red blood cell (RBC) transfusion can be lifesaving, it also can be life-threatening if an acute hemolytic transfusion reaction (AHTR) occurs. The resultant, intravascular hemolysis may lead to disseminated intravascular coagulation (DIC), shock, and acute renal failure (ARF) [1]. AHTR typically occurs early during transfusions, after the administration of as little as a few milliliters of incompatible blood, due to ABO incompatibility [1].

In a review of deaths related to transfusions that occurred between 2013 and 2017, hemolysis accounted for 32 of 185 deaths (17%). Of these 32 deaths, approximately one-third (6% of the total) were due to ABO incompatibility, and the remaining two-thirds (11% of the total) were due to non-ABO reactions [1].

Intravascular hemolysis results in the release of circulating free hemoglobin. With massive hemolysis, haptoglobin stores drop and hemoglobin then dissociates into α-β dimers resulting in hemoglobinuria, hemoglobin cast formation, heme uptake by proximal tubular cells, acute tubular necrosis, and filtration failure [2].

Historically, ABO-incompatible blood transfusions were the most common cause of hemolysis-associated AKI; however, these are now rare due to improved blood banking practices. In the current practice, among patients with hemolysis, AKI is rarely attributed to isolated hemolysis and is much more likely to be multifactorial [3].

Here, we encountered an AHTR in an obstetric patient that was the result of an ABO-incompatible blood transfusion before a cesarean section. The intravascular hemolysis immediately resulted in severe and prolonged ARF, which led to the patient requiring kidney replacement therapy and a long hospital stay. This case report examines one such presentation in line with the SCARE guidelines [4].

## 2 Presentation of case

A 29-year-old female of blood group A positive with anuria, edema, and elevated kidney function was transferred to our emergency department. In the previous three days and before the cesarean section, the patient described having flank and back pain, generalized aches, headache, fever, and 1–1.5L of bloody urine followed by anuria, immediately after the transfusion of 100–200 mL from a blood unit. Thereafter, an emergency cesarean section was performed and the infant was intact. The patient's serum creatinine (S-Cr) level was 0.8 mg\dL but it rose suddenly postoperatively to 8.8 mg\dL and the examination of the transfusion unit showed it to be blood group AB positive.

**Fig. 1:**
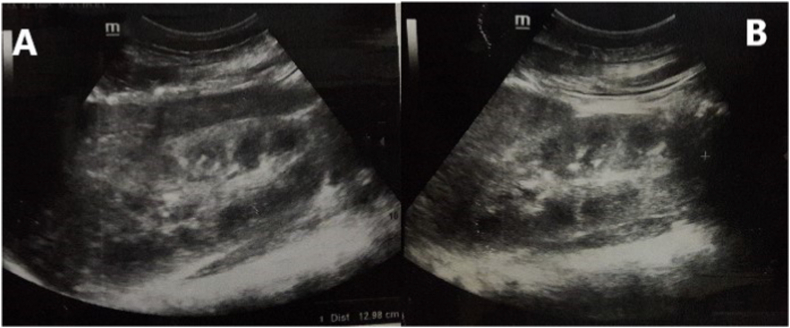
Renal ultrasound shows (A) right kidney diameter 12.9 cm, (B) lift kidney diameter 12 cm, and both kidneys show increase in cortical echogenicity, reduce corticomedullary differentiation and pyramids enlargement.

The physical examination in the emergency was as follow: blood pressure was 130/80, respiratory rate was 20/min, heart rate was 78 beats/min, temperature was 37.2 C, oxygen saturation was 96%, and she had grade II edema in lower extremities and jaundice in the sclera. Laboratory tests on admission (Table 1) showed findings that corresponded with intravascular hemolysis and ARF. Kidney ultrasound showed an increase in cortical echogenicity, a loss of corticomedullary differentiation, an enlargement of pyramids, and inferior vena cava diameter of 12 mm, with collapsibility in respiration (Fig. 1).

**Table 1:** Laboratory tests and procedures.

Date	Laboratories								UO\ 24h	Notes
10\Oct (admission)	WBC	14.2	DB	1.4	aPTT	13	Urine		100mL	
	HB	8.2	Na	134	INR	1	PH	5.5		
	PLT	84	K	4.1	PH	7.29	WBC	200		
	Ur	166	P	7.3	PCO_2_	23	RBC	2000		
	Cr	9.5	Ca	7.2	PO_2_	73	cells	150		
	LDH	800	UA	10	HCO_3_	11	HB	++++		
	TB	2.4	PT	100%	SO_2_%	93%	Protein	+		
12-13\Oct	HB	6.6	PLT	149	Cr	11.8	Ur	215	250–500 mL	RBCs unit
	LDH	331	TB\ DB	0.8\ 0.7	PH	7.30	HCO_3_	15		HD-session^a^
18-19\Oct	HB	8	PLT	321	Cr	12.6	Ur	175	700 mL	HD-session^b^
	PH	7.37	HCO_3_	15	PO_2_	34	SO_2_%	65%		
26-27\Oct	HB	7.5	Ur	218–199	Cr	6.7			3–4 L	Diuresis phase
2\Nov	HB	7.5	Ur	115	Cr	5.5			2 L	
5\Nov (discharge)	HB	7.4	Ur	85	Cr	4.1			2 L	
23\ Nov	HB	6.2	Ur	41	Cr	1.3	MCV	74		Iron supplements

WBC; white blood count, HB; hemoglobin, PLT; platelets, Ur; urea, Cr; creatinine, TB; total bilirubin, DB; direct bilirubin, LDH; Lactate dehydrogenase, Na; sodium, K; potassium, P; phosphorus, Ca; calcium, UA; uric acid, PT; prothrombin time, INR; international normalized ratio, aPTT; activated partial thromboplastin time, RBC; red blood cells, UO; urinary output, HD; hemodialysis.

a Two hemodialysis sessions due to uremia.

b Two hemodialysis sessions due to acute pulmonary edema.

The patient was treated with fluids, sodium bicarbonate, and diuretics. Hemodialysis (HD) was performed four times during their hospital stay. Two HD sessions were required to treat early severe uremia, and two HD sessions were required to treat acute pulmonary edema that developed due to non-compliance of the patient with fluid restriction (Table 1). Kidney function improved very slowly and returned to near normal after six weeks (Table 1).

## 3 Discussion

AHTR is a medical emergency with an estimated incidence of one per 70,000 blood product transfusion and an estimated mortality rate of five per 10 million RBC unit-transfusions [5,6]. AHTR usually occurs due to ABO incompatibility, which is most often the result of a clerical or procedural error [7]. The abundance of antigens on the RBC surface also influences the severity of the reaction, and this antigen density varies significantly for different RBC antigens. For example, ABO antigens are present at approximately 200,000–800,000 copies per cell [8].

It is important to note that the classic presentation triad of fever, flank pain, and red or brown urine is rarely seen. However, these symptoms may not be immediately apparent if the patient is under anesthesia; in such cases, oozing from venipuncture and dark urine due to DIC and hemoglobinuria, respectively, may be the only findings [1].

In this case, the patient displayed the classic triad a few minutes after a RBC transfusion, and fortunately, the infant was saved by the performance of an immediate cesarean section. This case was the second obstetric patient, admitted to our hospital, in the past few months, with ARF resulting from an AHTR induced by an ABO-incompatible blood transfusion.

The overall prognosis for patients with heme-induced AKI is favorable; most survivors will recover to a level of normal or near-normal kidney function [9]. However, this favorable prognosis does not mean that the risk of complications and sequelae of ARF is eliminated, in fact, large population-based studies have demonstrated a considerable risk of progressing to chronic kidney disease for patients who survive an episode of ARF, especially those who require dialysis [10].

Our patient incurred severe complications of ARF, that required dialysis, a long hospital stay, and a prolonged recovery period. In the context of hemolysis-associated AKI; although the use of dialysis for the removal of hemoglobin or uric acid to prevent or alleviate ARF has not been proven, it may be necessary in some cases to treat metabolic disturbances [9].

A 2015 study reported the prevalence of preoperative anemia in women undergoing gynecological surgery was 23.9% and associated with adverse postoperative outcomes; however, this risk did not appear to be reduced by perioperative transfusion [11,12]. In addition to the risks and potential long-term complications of RBC transfusion, optimal transfusion practice tends to prefer a restrictive transfusion strategy; while providing sufficient RBC to maximize clinical outcomes [13].

## 4 Conclusion

The complications of RBC transfusion can be disastrous. They can result in a long hospital stay and incur high costs, as was the situation of our patient who experienced ARF for several weeks. This case demonstrates the need for medical staff to reserve blood transfusions for obvious indications, to repeat the blood type, and to confirm the recorded compatibility of the patient and the blood unit before transfusion. Also, medical staff should always monitor the patient's symptoms during the transfusion process, to early recognize these severe conditions and to administer effective treatments as soon as possible.

## Consent

Written informed consent was obtained from the patient for publication of this case report and accompanying images. A copy of the written consent is available for review by the Editor-in-Chief of this journal on request.

## Ethical approval

Written informed consent was obtained from the patient for publication of this case report and accompanying images, in line with local ethical approval requirements and in accordance with the helsinki declaration.

## Sources of funding

This research did not receive any specific grant from funding agencies in the public, commercial, or not-for-profit sectors.

## Author contribution

Dr. Mohammad Alsultan wrote the manuscript, searched the literature, treat and follow up the patient and submitted the article.

## Registration of research studies

N/A.

## Guarantor

The corresponding author is the guarantor of this manuscript.

## Provenance and peer review

Not commissioned, externally peer-reviewed.

## Declaration of competing interest

The author declares that they have no conflicts of interest regarding this study.
